# In Vivo Partial Cell Reprogramming With Yamanaka Factors Reduced Ischemic Brain Damage in Aging Mice

**DOI:** 10.1111/acel.70776

**Published:** 2026-10-23

**Authors:** Paula R. Mallavibarrena, Gerardo Martín‐López, Jorge Rodríguez‐Morales, Jesús Ávila, Félix Hernández, María José Pérez‐Álvarez

**Affiliations:** ^1^ Departamento de Biología (Fisiología Animal), Facultad de Ciencias Universidad Autónoma de Madrid (UAM) Cantoblanco Madrid Spain; ^2^ Centro de Biología Molecular Severo Ochoa, CSIC/UAM Universidad Autónoma de Madrid Cantoblanco Madrid Spain; ^3^ Center for Networked Biomedical Research on Neurodegenerative Diseases (CIBERNED) Instituto de Salud Carlos III Madrid Spain; ^4^ Instituto Universitario de Biología Molecular (IUBM) UAM Cantoblanco Madrid Spain

**Keywords:** glia, myelin, neuroprotection, oligodendrocytes, partial reprogramming, pMCAo, stroke, Yamanaka factors

## Abstract

Aging is associated with functional decline and reduced regenerative capacity in the brain, is also the strongest risk factor for ischemic stroke, which remains a leading cause of mortality and long‐term disability worldwide, and is associated with impaired functional recovery. Here, we investigated whether short‐term partial cellular reprogramming through cyclic expression of Yamanaka factors (OSKM) in a Tet‐ON mouse model under Rosa‐26 promoter has any impact on ischemic damage in aged mice. To assess ischemic resilience, mice underwent permanent middle cerebral artery occlusion (pMCAo). Forty‐eight hours after ischemia, OSKM‐induced mice displayed significantly smaller damaged area compared with wild‐type controls, accompanied by improved neurological scores and enhanced performance in the tightrope test, suggesting preserved sensorimotor abilities. Notably, myelin integrity was preserved in OSKM‐treated mice, as shown by Black‐Gold staining and stable MAG and MBP expression, whereas wild‐type mice exhibited pronounced demyelination. Given the role of oligodendrocytes in myelin maintenance, we quantified the abundance of their different populations. OSKM induction significantly increased total oligodendrocytes (Olig2^+^) and oligodendrocyte precursor cells (PDGFRα^+^) following ischemia, suggesting an enhanced reparative response. Moreover, partial reprogramming preserved neuronal density in the ischemic cortex and prevented the marked astrocytic activation observed in wild‐type mice, while microglial responses remained comparable across genotypes. These findings demonstrate that cyclic partial reprogramming in aged mice reduces ischemic damage, highlighting OSKM induction as a promising strategy for enhancing brain resilience during cerebrovascular accidents.

## Graphical abstract

Short‐term cyclic partial reprogramming with Yamanaka factors protects aged mice from ischemic brain injury, preserving cognitive function, myelin integrity, and neuronal density, supporting partial reprogramming as a promising neuroprotective intervention in aging.

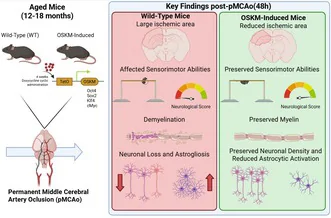

## 1 Introduction

The cerebrovascular accident (CVA), commonly known as stroke, represents the primary cause of long‐term disability and the second leading cause of mortality globally (Martin et al. 2025). Approximately 87% of strokes are classified as ischemic and exhibit a significant correlation to aging (Springer et al. 2024). Indeed, aging is the primary risk factor for CVA (Roy‐O'Reilly and McCullough 2018), and older stroke patients typically experience poorer prognoses and functional recovery compared to their younger counterparts (Fisher et al. 2009). In recent years, the global demographic trend has experienced a progressive aging of the population, which is more evident in developing countries. Therefore, it is estimated that the incidence of stroke will increase in the coming years (Zhao et al. 2025).

Ischemic stroke occurs when blood flow to the brain is reduced or impaired, usually by a thrombus or an atherosclerotic plaque. This disruption triggers the “ischemic cascade” that involves, among others, cell death, inflammation, oxidative stress, and demyelination (Dirnagl et al. 1999; Hernández et al. 2021). Currently, the sole therapeutic approach employed to mitigate the harmful effects of brain ischemia includes reperfusion through thrombolytic agents (such as tPA) or mechanical thrombectomy (Jia et al. 2024). These methods present a limited window of opportunity for their application, since they can promote hemorrhage in patients (Jia et al. 2024; Verhoeven et al. 2022). In addition, they fail to effectively prevent the long‐term neurodegeneration associated with ischemia (Yang et al. 2025). Furthermore, they may exacerbate the initial damage, since they can induce ischemia/reperfusion injury, prompted by the sudden restoration of blood supply to the ischemic region (Kumar Saini and Singh 2024; Yang et al. 2025). Therefore, it is necessary to search for new therapeutic strategies to reduce the negative effects of ischemia and reperfusion in the brain.

Aging is a natural process characterized by the gradual accumulation of molecular and cellular damage, leading to a decline in physical and mental capacity, and an increased risk of disease (López‐Otín et al. 2023). One of the main affected organs is the brain, which experiences significant alterations in the function and plasticity of neuronal circuits, resulting in motor and cognitive impairment (Antón‐Fernández et al. 2025; Budygin et al. 2025). As neurons age, they lose synaptic connections, show reduced ability for transmitting nerve impulses, and undergo metabolic alterations. This raises the question of whether aging‐related effects can be reversed in highly specialized cells such as neurons. A promising new approach to enhance cellular function and restore tissue homeostasis during aging is the overexpression of Yamanaka factors (YFs), also known as OSKM: Oct4, Sox2, Klf4, and c‐Myc, in aged mice. In vivo cell reprogramming was successfully conducted for the first time using a Rosa26‐rtTA‐TetO‐OSKM mouse model through continuous administration of doxycycline. This approach led to a significant induction of teratomas, the presence of induced pluripotent stem cells (iPSCs) in the blood capable of forming embryonic structures, and a notably reduced life expectancy in mice (Abad et al. 2013). To prevent these effects, in vivo reprogramming through cyclical and controlled expression of YFs has shown promising results in improving various peripheral diseases related to aging (Ocampo et al. 2016). Thus, partial reprogramming in vivo with the YFs has been shown to enhance regeneration and reduce age‐related damage in multiple tissues, including skeletal muscle (Browder et al. 2022; Wang et al. 2021), liver (Hishida et al. 2022), retina (Lu et al. 2020), skin (Doeser et al. 2018), and cardiovascular tissue (Chen et al. 2021; Cheng et al. 2022), by decreasing cellular senescence and restoring epigenetic markers of youth. The cyclical and controlled expression of YFs prevents complete dedifferentiation while promoting repair, making it a promising strategy for multi‐organ rejuvenation (Nakatsukasa et al. 2025; Ocampo et al. 2016), including the brain (Rodríguez‐Matellán et al. 2020; Xu et al. 2024). Also, cyclical overexpression of YFs in two mouse models of Alzheimer's disease results in an improvement of the main pathological hallmarks (Antón‐Fernández et al. 2025; Shen et al. 2024). These results suggest that partial reprogramming could be a new therapeutic strategy to reduce aging‐related neurodegeneration.

The impact of cellular reprogramming in an ischemic context has not been deeply investigated, particularly in elderly mice. This gap in research is significant, as aging plays a crucial role in the increased incidence of stroke in humans (Fisher et al. 2009; Roy‐O'Reilly and McCullough 2018). The continuous induction of YFs expression in situ by intraventricular perfusion of doxycycline for 7 days after global ischemia, in young mice (8–16 weeks old), facilitates functional recovery and enhances neovascularization of the ischemic area (Seo et al. 2016). Intraventricular injection of a viral vector containing OSKM factors in a model of neonatal chronic hypoxic–ischemic brain injury improved long‐term memory, decreased anxiety, and enhanced hippocampal neurogenesis (Wi et al. 2016). However, the effects of in vivo brain cell reprogramming in elderly mice in an ischemic context have not been characterized yet.

Given that the prevalence and outcomes of stroke are closely related to aging, we aimed to explore the effects of partial reprogramming in aged mice. Additionally, we aimed to assess whether cyclic overexpression of YFs over 4 weeks could be an effective strategy that might mitigate ischemic damage in the central nervous system. The results of this study contribute to a deeper understanding of the potential role of cell reprogramming in aging in the context of stroke.

## 2 Materials and Methods

### 2.1 Animals

A total of 30 C57BL/6 mice (16 male and 14 female), aged 12–18 months, were maintained in the animal facility of the Centro de Biología Molecular Severo Ochoa (CBMSO). Animals were housed in a specific pathogen‐free colony under standard laboratory conditions, in accordance with European Community guidelines and all applicable institutional and national regulations for the care and use of laboratory animals. All experimental procedures were reviewed and approved by the CBMSO and UAM Ethics Committees and authorized by Comunidad de Madrid (PROEX_042.5_21 and PROEX 333.4_24). Mice were group‐housed (4–5 per cage) with *ad libitum* access to food and water, in a temperature‐controlled environment with a 12:12 h light/dark cycle.

The transgenic mouse line i4F‐B, used in this study, was previously generated and characterized by (Abad et al. 2013). These mice harbor the reverse tetracycline‐controlled transactivator (rtTA), inserted into the ubiquitously expressed Rosa26 locus, along with a single‐copy lentiviral, doxycycline‐inducible polycistronic cassette encoding the four murine Yamanaka reprogramming factors (Oct4, Sox2, Klf4, and c‐Myc). The transgene is integrated within an intronic region of the *pparg* gene. For the present work, we used heterozygous transgenic animals carrying one copy of the inducible cassette (hereafter referred to as Yamanaka, yk) and wild‐type littermates from the same colony (hereafter referred to as wild‐type, wt).

### 2.2 In Vivo Induction of Yamanaka Factors

Yamanaka factors expression was induced in reprogrammable mice by administering doxycycline (2 mg/mL; Sigma) in the drinking water. A cyclic induction protocol was employed, which has been previously reported not to affect mortality (Rodríguez‐Matellán et al. 2020). This consisted of weekly cycles of 3 days of doxycycline treatment followed by 4 days of withdrawal, applied for four consecutive weeks prior to surgery. Doxycycline was administered to all the mice used in this study, including wt.

### 2.3 Ischemia Induction

Animals were randomly assigned to either the sham (*n* = 16) or permanent middle cerebral artery occlusion (pMCAo) (*n* = 14) groups. The distribution of mice used according to sex, genotype, and age is summarized in the Supporting Information (Table S1). Both sexes were included across groups, ensuring a balanced sex distribution within each experimental condition. For age, the distribution among genotypes was consistent within each condition: Sham ranged from 16 to 18 months, and pMCAo from 12 to 17 months.

The pMCAo procedure was performed following the Koizumi method 6 h after doxycycline withdrawal (Koizumi et al. 1986; Morris et al. 2016). Anesthesia was induced with 3% isoflurane and subsequently maintained at 1%–1.2% throughout the surgical procedure. A midline cervical ventral incision was made to expose the right common carotid artery (CCA). The CCA and external carotid artery (ECA) were permanently ligated, after which an arteriotomy was performed in the CCA near its bifurcation. A silicon‐coated 6‐0 monofilament (Doccol 602112PK10) was inserted through the arteriotomy and advanced into the right internal carotid artery until reaching the origin of the middle cerebral artery (MCA). The filament was secured with a ligature and left in place until the animals were euthanized 48 h post‐surgery. Sham‐operated animals underwent identical surgical manipulations except for filament insertion. To ensure analgesia, all animals received topical skin anesthesia with lidocaine/prilocaine cream (25 mg/g; EMLA, AstraZeneca) and were administered buprenorphine (0.3 mg/mL, subcutaneously) prior to the procedure.

### 2.4 Behavioral Testing

To evaluate whether partial reprogramming affected cognitive and motor function in sham‐operated animals, locomotor activity was assessed 24 h after surgery, using the Open Field Test (OFT). Mice were allowed to freely explore a 40 × 40 × 60 cm arena for 10 min while being video recorded. Recordings were analyzed using ANY‐maze software to quantify total distance traveled (m), average speed (m/s), and resting time (s) as measures of motor activity. Time spent in the center of the arena was used as an index of anxiety‐like behavior (Seibenhener and Wooten 2015).

To determine the impact of partial reprogramming on ischemic injury in pMCAo animals, we conducted additional behavioral tests prior to surgery and at 6‐, 24‐, and 48 h following the procedure. Neurological deficits were assessed using a seven‐point scoring system previously described (Yrjänheikki et al. 2005), which enabled the calculation of the Ischemic Damage Index (IDI). Motor coordination, balance, and global locomotion, including all four limbs, were further evaluated using the Beam Walking Test. Mice were placed at one end of a narrow beam, and their ability to traverse it was assessed using a five‐point scoring scale adapted from (Chen et al. 2019): 0—the mouse could not grasp the beam and fell; 1—the mouse grasped the beam and stayed on it for 1 min but could not move along it; 2—the mouse maintained balance but could not cross the beam; 3—the mouse moved along the beam but feet slipping occurred; and 4—the mouse moved freely along the beam and reached the other end without any difficulty. Additionally, the tightrope test was employed to assess motor coordination and grip strength of forelimbs (more affected in MCAo models). Mice were placed on one end of a 60‐cm rope, holding on with their forepaws. The maximum test duration was 60 s, and performance was scored from 0 (minimum) to 20 (maximum) according to the validated scoring system (Doeppner et al. 2011) based on time spent on the rope and whether the animal reached the platform.

### 2.5 Sacrifice and Tissue Processing

For histological analyses, mice were anesthetized with an intraperitoneal injection of pentobarbital (Dolethal, 60 mg/kg body weight) after 48 h of surgery. Next the mice were transcardially perfused with ice‐cold phosphate‐buffered saline (PBS), followed by 4% paraformaldehyde (PFA) in PBS (pH 7.4). Brains were post‐fixed overnight at 4°C in 4% PFA and subsequently cryoprotected by immersion in 10% sucrose for 24 h and 30% sucrose for 48 h. Following cryoprotection, brains were embedded in Tissue‐Tek O.C.T. compound (Sakura) and stored at −20°C until sectioning. Coronal sections (25 μm thickness) spanning Bregma −0.95 to +1.20 (Paxinos and Watson 2019) were obtained using a Leica CM1520 cryostat and mounted onto Superfrost Ultra Plus slides (Thermo Scientific, Germany). Sections were stored at −20°C until further processing.

### 2.6 Nissl and BlackGold II Staining

To quantify the ischemic area, equivalent coronal brain sections from mice were stained using the Nissl method as described by (Villa González et al. 2020). Brain sections were sequentially rehydrated through decreasing ethanol concentrations and stained with cresyl violet (2% in acetate buffer; Sigma). Following staining, samples were dehydrated through increasing ethanol concentrations, cleared in xylene (PanReac), and mounted with DEPEX mounting medium (PanReac). The damaged area was delineated using morphological criteria previously defined by (Mateos et al. 2016). The images of entire right hemispheres were obtained using the Process Manager tool (CellSensDimension, Olympus) on an Olympus BX63 brightfield microscope. The percentage of damaged area was calculated as the percentage of ischemic surface relative to the total hemisphere area.

Myelination degree was assessed in brain sections using Black‐Gold II staining (Biosensis), following the manufacturer's instructions. Sections were rehydrated in distilled water for 2 min, incubated in Black‐Gold II solution at 65°C for 12 min, rinsed in distilled water, and subsequently treated with 1% sodium thiosulfate. Samples were then dehydrated and mounted with DEPEX medium (PanReac). For quantification, six images per brain section were acquired from the ischemic region (cerebral cortex layers I–III) in the right hemisphere, analogously to (Martín‐Lopez et al. 2024), using an Olympus BX63 light microscope equipped with an Olympus DP74 camera. Images were analyzed with ImageJ (Schindelin et al. 2012), and myelination was quantified as the percentage of stained area determined by color threshold.

### 2.7 Immunofluorescence (IF) and Quantifications

For immunofluorescence analyses, sections were first incubated in 0.1 M glycine in PBS (pH 8.5) for 30 min to reduce autofluorescence. Tissue permeabilization was performed in 0.25% Triton X‐100 (Tx‐100) in PBS for 15 min, followed by incubation in blocking buffer (1% horse serum, 1% BSA in PBS containing 0.1% Tx‐100) for 1 h at room temperature (RT). For certain primary antibodies, antigen retrieval was required and performed by incubating the slides at 85°C in 10 mM citrate buffer (pH 6.0) for 30 min. For optimal detection of Klf4 and OCT4, brain sections were pretreated with 2 M HCl for 10 min at 35°C. Primary antibodies were diluted in blocking solution at the appropriate concentrations and incubated overnight at 4°C. After three washes in PBS, sections were incubated for 2 h at RT with the corresponding Alexa Fluor–conjugated secondary antibodies (1:500; ThermoFisher). This was followed by three additional PBS washes, incubation with DAPI (1:5000; Millipore) for 10 min, and three final washes. Slides were mounted with Fluoromount‐G (SouthernBiotech) to preserve fluorescence. The following primary antibodies were used: mouse anti‐MAG (1:200; Millipore, MAB1567), rat anti‐MBP (1:300; Millipore, MAB386), rabbit anti‐PDGFRα (1:200; Abcam, ab20349), mouse anti‐BCAS1 (1:500; Santa Cruz, SC‐136342), mouse anti‐CC1 (1:200; Abcam, ab16794), rabbit anti‐Olig2 (1:200; Millipore, AB9610), rabbit anti‐NeuN (1:1000; Merck, ABN78), chicken anti‐GFAP (1:500; Abcam, ab4674), guinea pig anti‐Iba1 (1:750; lab clinics, HS‐234004), goat anti‐Klf4 (1:250; R&D system, af3158), and rabbit anti‐Oct4 (1:500; R&D system, SAB5700848).

For NeuN^+^ cell counting, three consecutive images of each slice were taken from cortical layers II–III (see Figure 3C). For PDGFRα^+^, BCAS1^+^, CC1^+^, and Olig2^+^ cell counting, three consecutive images were taken of the cortical layer V–VI of the damaged area of the right hemisphere (Martín‐Lopez et al. 2024). PDGFRα^+^ and BCAS1^+^ cells were manually counted using ImageJ cell counting tool (Schindelin et al. 2012). We used the “Analyze Particles” tool of Image J (Schindelin et al. 2012) to quantify Olig2^+^ and CC1^+^ cells with a size = 200‐Infinity and circularity = 0.08–1. The results are expressed as the total number of cells from all three fields per mm^2^. Images were obtained with a 20× lens on a Zeiss LSM900 confocal microscope. For GFAP and Iba1 analysis, three images of each slice were taken from the penumbra region of the right hemisphere (see Figure 4E). Cells were counted using the “Analyze Particle” tool with a size of 150‐infinity pixels on images obtained with a 10× lens on an Olympus BX63 fluorescence microscope. For MBP and MAG staining, six photographs were taken of each brain section of the cerebral cortex layers I–III (similar to the selected region for Black gold analysis) of the right hemisphere using an Olympus BX63 fluorescence microscope, represented as the percentage of stained area by adjusting the color threshold.

For illustrative purposes, the same representative microphotograph was used in Figures 3C and 4E. Different regions of interest (ROIs) from this image were selected and annotated to illustrate the distinct quantitative analyses performed in each figure. The image was not used to represent different experimental conditions.

### 2.8 Statistics

Data are presented as mean ± standard error of the mean (SEM) in bar plots, with individual animal values represented in corresponding dot plots. Each animal value was obtained from the quantification of a minimum of three serial slices per animal and three to six fields of view (FOV) per brain slice. The number of FOVs per brain slice was determined according to the variability of the measured parameter to ensure sufficient statistical power. Statistical analyses were conducted using GraphPad Prism 10. All experiments, including data acquisition and analysis, were performed in a randomized order by blinded investigators. Specifically, investigators were blinded to the experimental groups during behavioural testing and microscopic examination of the samples. The Shapiro–Wilk test was applied to assess the normality of data distributions. For comparisons involving more than two groups, a two‐way analysis of variance (ANOVA) followed by Bonferroni post hoc correction was used. For comparisons between two groups, unpaired *t*‐tests were applied. A significance threshold of *p* < 0.05 was considered for all statistical tests.

An AI‐assisted language editing tool (Grammarly free version) was used to improve the clarity and grammar of the manuscript. The tool did not influence the scientific content, analysis, or conclusions of the manuscript.

## 3 Results

### 3.1 Impact of Partial Cell Reprogramming on Motor Capacity

First, we aimed to determine whether partial cell reprogramming for 4 weeks induced any behavioral changes in elderly mice (12–18 months old). We used the open field test (Figure 1A) to assess motor activity (distance, mean speed, and resting time) as well as anxiety levels (measured by the time spent in the center of the box).

**FIGURE 1:**
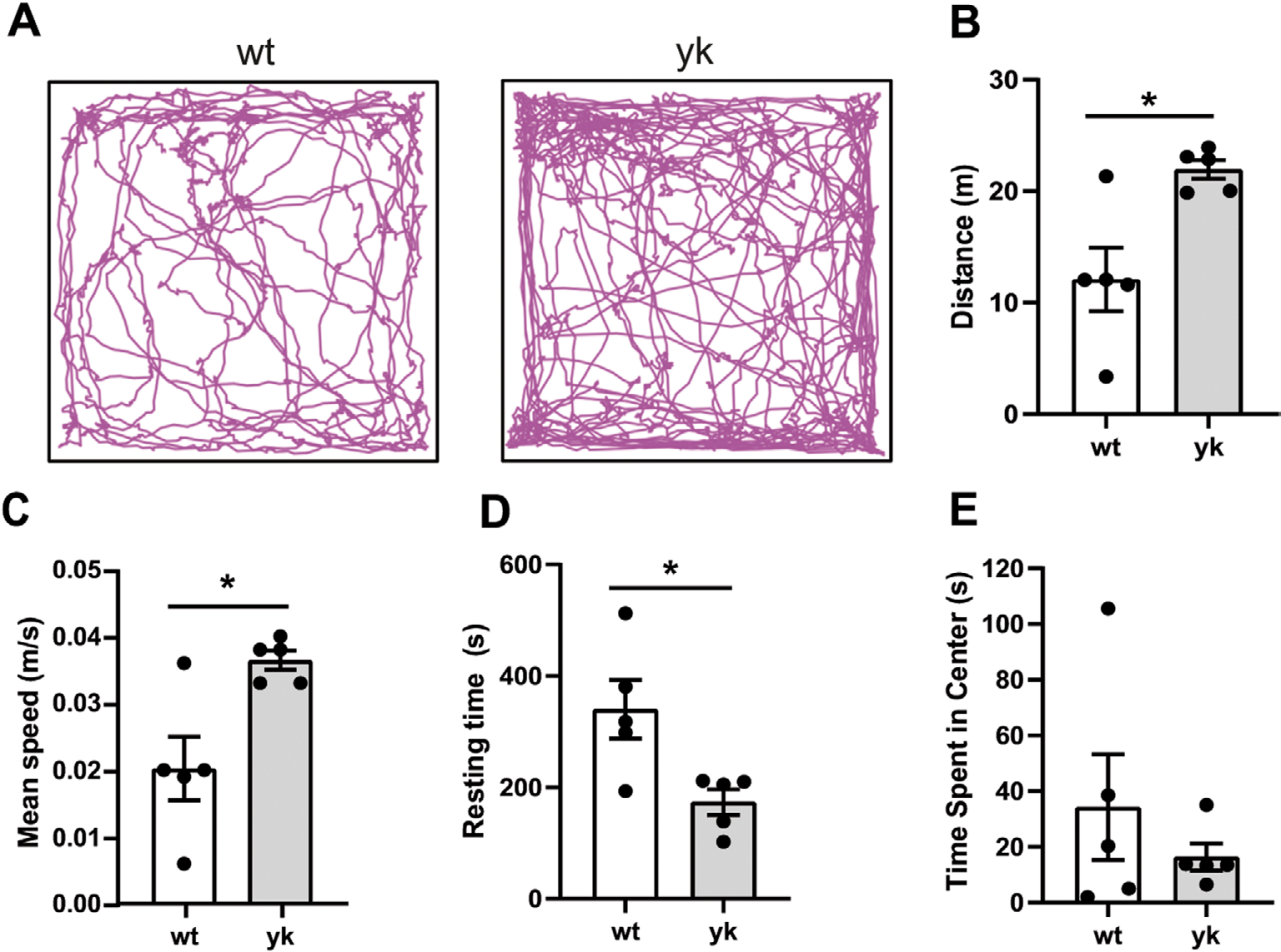
Partial cellular reprogramming impact on behavior. (A) Representative track plots from the open‐field test for each genotype. (B) Total distance traveled. (C) Mean velocity. (D) Resting time. (E) Time spent in the center zone. The open‐field test duration was 600 s. Data are presented as mean ± SEM (*n* = 5). Statistical significance was determined using an unpaired two‐tailed t‐test. *p < 0.05. wt, wild‐type; yk, transgenic mice with induced expression of Yamanaka factors.

Regarding motor activity, we observed that yk‐mice traveled a greater distance (21.96 ± 0.84 m compared to 12.08 ± 2.84 m; *p* < 0.05; Figure 1B) and had a higher average speed (0.036 ± 0.001 m/s compared to 0.020 ± 0.004 m/s; *p* < 0.05; Figure 1C) than wt‐mice. Additionally, yk‐mice remained motionless for a shorter time (173.5 ± 22.52 s compared to 340 ± 52.49 s; *p* < 0.05; Figure 1D) compared to wt. We can conclude that YFs' induction, during 4 weeks in aged mice, results in a more active phenotype compared to that of wt mice.

To determine if these improvements in motor activity could be linked to anxiety levels in transgenic mice subject to partial cell reprogramming, we measured the average time that the mice spent in the center of the box. No significant differences were observed between the experimental groups (34.28 ± 18.95 s wt group and 16.42 ± 4.84 s yk group).

To confirm the expression of YFs following 4 weeks of cyclic doxycycline administration in yk‐mice, and to elucidate the affected cellular population, we conducted a double immunofluorescence assay to visualize the expression of the Klf4 and Oct4 proteins in conjunction with specific cellular markers for neurons (NeuN), astrocytes (GFAP), microglia (Iba1), and oligodendrocytes (Olig2). Notably, *Oct4* and *Klf4* occupy the first and third positions in the gene order of the OSKM transgene. Our results demonstrated that all analyzed cellular populations express Oct4 and Klf4 (Figure S1).

### 3.2 Cyclic Overexpression of Yamanaka Factors Before pMCAo Reduces the Damaged Area and Partially Improves the Neurological Score of the Animals

To evaluate the impact of partial reprogramming on ischemia, we employed two distinct approaches: a histological analysis using Nissl staining and a series of behavioral assessments (Neurological score, Beam walking, and Tightrope) conducted 6‐, 24‐, and 48‐h post‐surgery. The latest enables us to monitor the progression of the mice's condition.

The assessment of the damage area 48 h following pMCAo in the ipsilateral hemisphere (Figure 2B,C) indicated that the percentage of ischemic area in pMCAo‐wt animals was 31.5% ± 1.5% (*p* ≤ 0.0001 compared to the Sham‐wt group; Figure 2A). In contrast, yk mice exhibited significantly less damage, with an ischemic area of 24.9% ± 1.86% (*p* ≤ 0.05 compared to pMCAO‐wt group).

**FIGURE 2:**
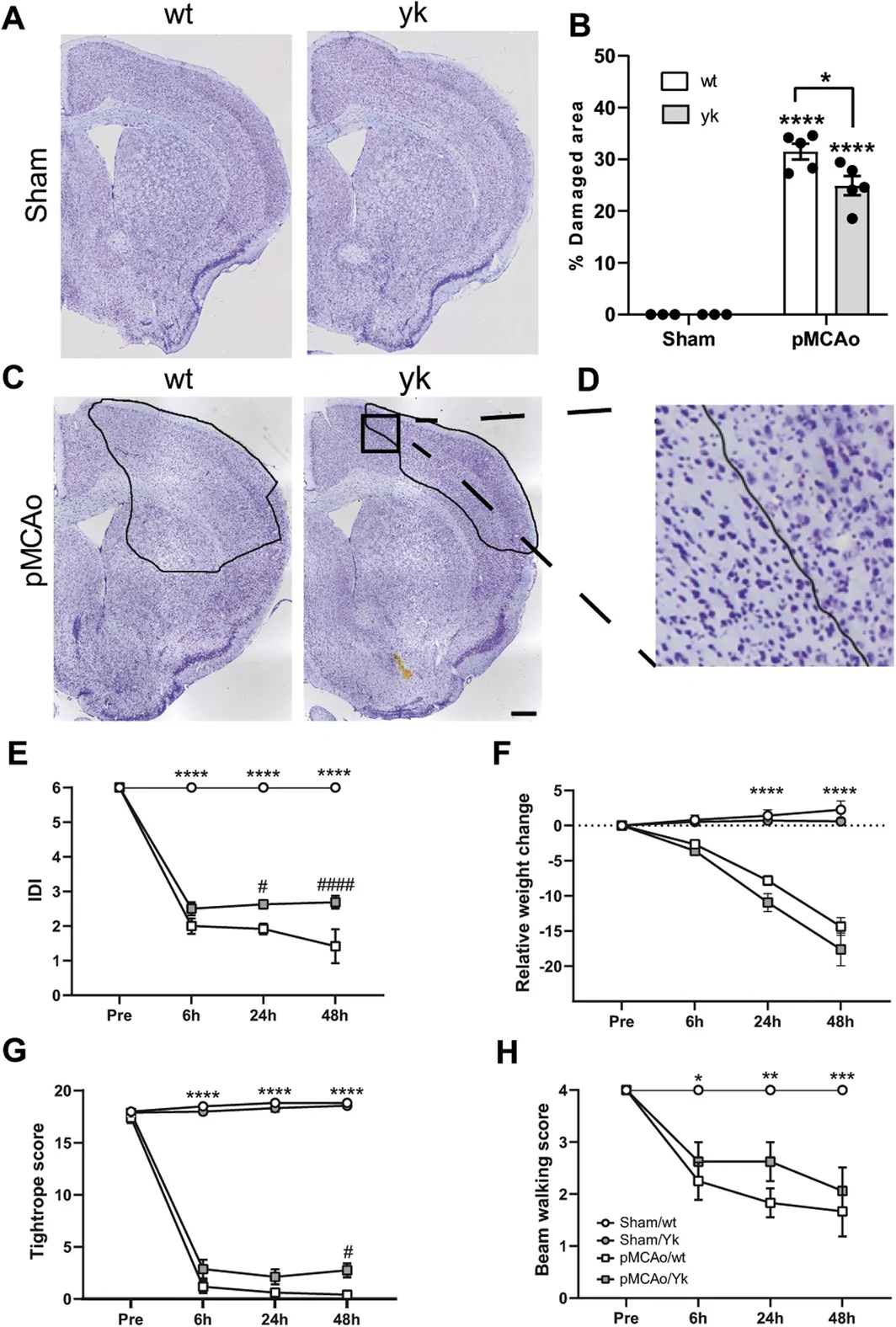
Cyclic induction of Yamanaka factors prior to ischemia reduces brain damage and improves neurological outcome after ischemia. (A) Representative low‐magnification montages of Nissl‐stained coronal brain sections (25 μm thick) from the right hemisphere of sham‐operated mice of each genotype. (B) Quantification of damaged area expressed as a percentage of the total hemispheric area in pMCAo mice of each genotype at 2 days post‐ischemia (*n* = 3 in sham groups and *n* = 5 in pMCAo groups). The white bars correspond to wt, and the gray bar corresponds to yk. (C) Representative low‐magnification montages of Nissl‐stained coronal sections (25 μm) from ischemic (pMCAo) mice at 2 days post‐occlusion; the solid line delineates the damaged area. Scale bar: 200 μm. (D) Higher‐magnification image of a Nissl‐stained section from a pMCAo‐yk mouse showing damaged (right) and preserved (left) tissue. (E) Ischemic damage index (IDI) was assessed using a seven‐point neurological scoring scale (0, lowest; 6, highest deficit). The data points for the groups Sham‐wt and Sham‐yk overlap entirely and therefore appear as a single set of symbols. (F) Body weight loss is expressed as a percentage of initial weight. (G) Tightrope performance was evaluated using a 20‐point scale (0, lowest; 20, highest score). (H) Beam‐walking performance was assessed using a 4‐point scale (1, lowest; 4, highest score). The data points for the groups Sham‐wt and Sham‐yk overlap entirely and therefore appear as a single set of symbols. Data from behavioral tests are presented as mean ± SEM (*n* = 6 Sham‐wt; *n* = 10 Sham‐yk; *n* = 6 pMCAo‐wt; *n* = 8 pMCAo‐yk). Statistical analysis was performed using two‐way ANOVA with Bonferroni *post hoc* correction. *p < 0.05; **p < 0.01; ***p < 0.001; ****p < 0.0001 for sham versus ischemic comparisons; ^#^ *p* < 0.05; ^####^ *p* < 0.0001 for comparisons between ischemic groups. IDI, ischemic damage index; pMCAo, permanent middle cerebral artery occlusion; wt, wild‐type; yk, transgenic mice with induced Yamanaka factor expression.

Neurobehavioral tests conducted 6 h after pMCAo demonstrated an IDI score of 2.0–2.5 in both experimental groups (wt and yk), indicating a successful ischemia induction (Figure 2E). There were no notable differences in weight loss between the genotypes (wt and yk) in either pMCAo group following ischemia (Figure 2F). After 24 h of ischemia, the IDI score for pMCAo‐wt remained consistent (1.9), while the pMCAo‐yk group exhibited a significant increase (2.62; *p* ≤ 0.05, compared to the pMCAO‐wt group). These differences between the two ischemic groups became even more pronounced 48 h post‐ischemia, with scores of 1.41 for pMCAo‐wt and 2.68 for pMCAo‐yk (*p* ≤ 0.0001) (Figure 2E). To determine whether the improvement in the IDI score observed in the yk group correlated with enhanced sensorimotor function associated with the parietal cortex, which is most affected by ischemia, we conducted two additional tests. The beam walking test revealed no significant differences between the wt and yk pMCAo mice (Figure 2H). However, the results from the tightrope test, which evaluates the motor function of forepaw limbs (notably impacted after MCAo), revealed a significant increase in pMCAo‐yk score compared to the pMCAo‐wt group at 48 h (0.4 pMCAo‐wt and 2.75 ± 0.67 pMCAo‐yk; *p* ≤ 0.05) (Figure 2G). This apparent contradiction shows that the effect of OSKM induction is task dependent. The beam walking test assessed global locomotion, including four limbs and balance (Tozzi et al. 2025), while the tightrope measured the use, coordination, and grip strength of forelimbs (Ruan and Yao 2020), which were more affected in the pMCAo model. These findings indicate that partial reprogramming improves the tasks associated with the ischemically damaged area, suggesting a minor ischemic effect on the yk mice at the behavioral level.

### 3.3 Partial Reprogramming Reduces the Loss of Neurons and Glia Response After 48 h of pMCAo Induction

One of the pathological consequences of ischemia is neuroinflammation, which is primarily mediated by the response of astrocytes and microglia (Hernández et al. 2021; Yang et al. 2025). The extent of the glial response closely correlates with the severity of ischemic damage (Anogianakis et al. 2024; Pérez‐Álvarez et al. 2012). In an ischemic event, the drastic reduction or absence of blood flow to the brain leads to a loss of neuronal function and subsequent cellular death. Our results (Figure 3A,B) show that cyclic Induction of Yamanaka factors for 4 weeks in aging mice results in significant protection against loss of neurons (Figure 3). In Sham animals (both wt and yk), neuronal density approaches nearly 300 cells/mm^2^, with no significant differences observed between the two genotypes (278 ± 10 cell/mm^2^ Sham‐wt group, and 310 ± 24 cell/mm^2^ Sham‐yk group). Following 48 h of pMCAo induction, wt animals exhibited a significant reduction in neuronal density (207.7 ± 30 cells/mm^2^; *p* < 0.05). In contrast, yk mice subjected to pMCAo maintained neuronal density comparable to that of the Sham groups (Figure 3A,B).

**FIGURE 3:**
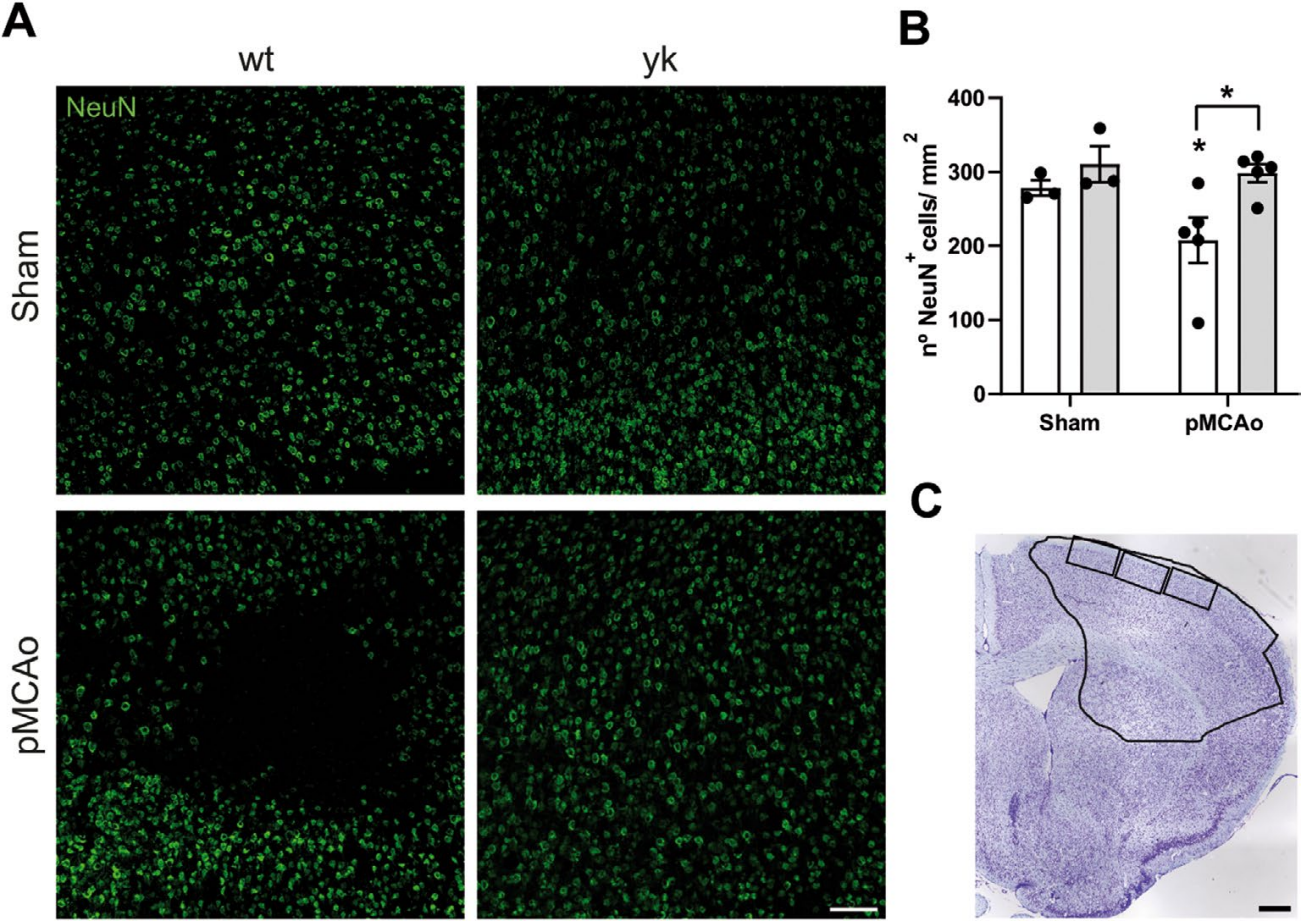
Partial reprogramming preserves neuronal density following cerebral ischemia. (A) Representative confocal images of the parietal cortex from coronal brain sections (25 μm thick) of sham‐operated and pMCAo mice of each genotype stained for NeuN. Scale bar: 20 μm. (B) Quantification of NeuN^+^ cell density (cells/mm^2^) in Sham and pMCAo mice 48 h after ischemia. White bars correspond to wt and the gray bars to yk genotypes. Data are presented as mean ± SEM (*n* = 3 in Sham groups and *n* = 5 in pMCAo groups). (C) Low‐magnification image of a Nissl‐stained coronal section of the right parietal cortex indicating the three fields of interest used for quantification in each brain section. The same image is also shown in Figure 4E as an anatomical reference for the regions analyzed. Scale bar: 200 μm. Statistical significance was determined by two‐way ANOVA with Bonferroni *post hoc* correction. *p < 0.05. NeuN, neuronal nuclei; pMCAo, permanent middle cerebral artery occlusion; wt, wild‐type; yk, transgenic mice with induced Yamanaka factor expression.

Accordingly, the glial response was assessed by measuring GFAP^+^ cells/mm^2^ (astrocytes) and Iba1^+^ cells/mm^2^ (microglia) in the penumbra region (Xu et al. 2025) (Figure 4). No significant differences were observed among the experimental groups regarding Iba1^+^ cells/mm^2^ (Figure 4B,D). Similarly, there were no notable differences between the Sham groups in terms of GFAP^+^ cells/mm^2^. However, a significant increase in the density of GFAP^+^ cells was observed in the pMCAO‐wt group compared to the Sham‐wt group (Figure 4A,C), with values of 228 ± 34 cells/mm^2^ for the Sham‐wt group and 445 ± 20 cells/mm^2^ for the pMCAO‐wt group (*p* < 0.05). Lastly, no variation was found between the pMCAo‐yk and Sham‐yk groups (Figure 4A,C).

**FIGURE 4:**
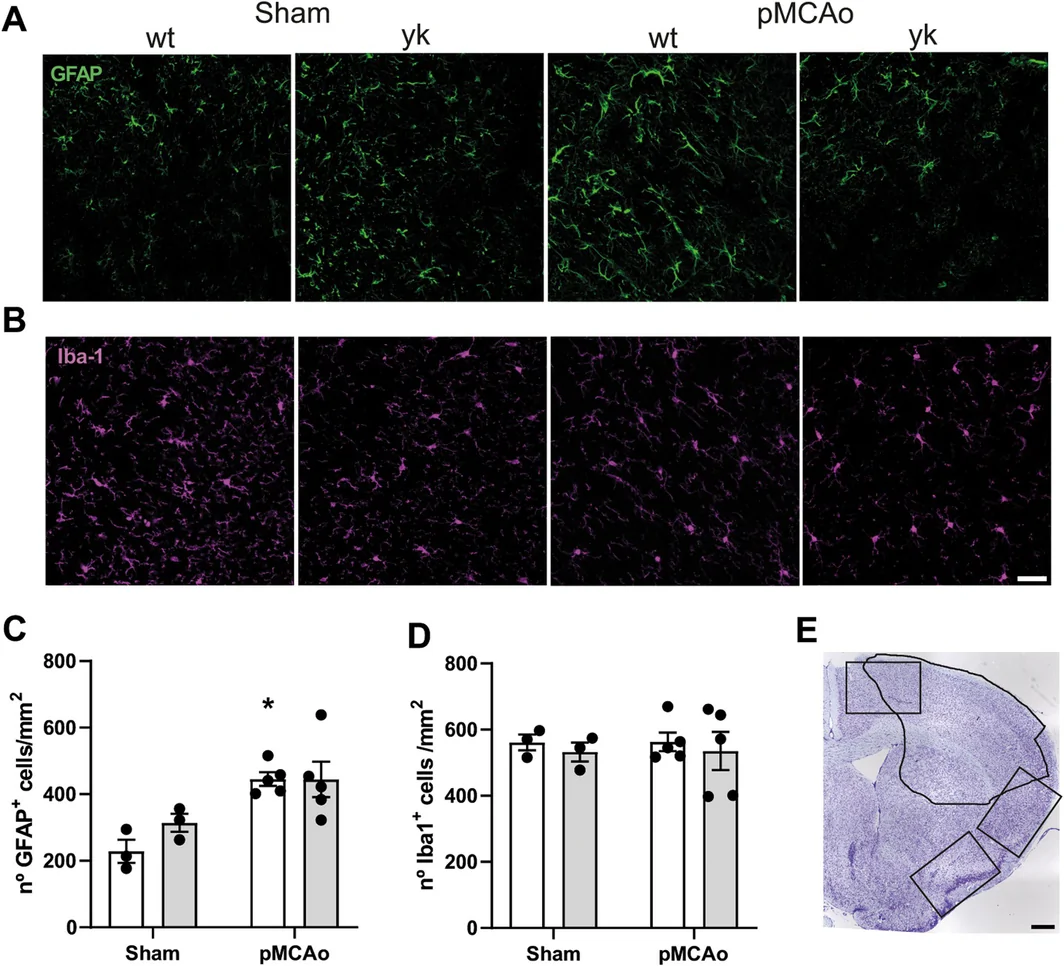
Cyclic induction of Yamanaka factors attenuates glial activation in the ischemic penumbra. (A, B) Representative confocal images of the penumbra region from coronal brain sections (25 μm thick) of Sham‐operated and pMCAo mice of each genotype stained for astrocytes (GFAP, green; A) and microglia (Iba1, magenta; B). Scale bar 20 μm (C, D) Quantification of GFAP^+^ (C) and Iba1^+^ (D) cell density (cells/mm^2^) in Sham and pMCAo mice. White bars correspond to wt and the gray bars to yk genotypes. (E) Low‐magnification image of a Nissl‐stained coronal section of the right parietal cortex indicating the three fields of interest used for quantification in each brain section. Scale bar: 200 μm. The same image as in Figure 3C is shown here as an anatomical reference for the regions analyzed. Data are presented as mean ± SEM (*n* = 3 in sham groups and *n* = 5 in pMCAo groups). Statistical analysis was performed using two‐way ANOVA with Bonferroni *post hoc* correction. *p < 0.05. GFAP, glial fibrillary acidic protein; Iba1, ionized calcium‐binding adapter molecule 1; pMCAo, permanent middle cerebral artery occlusion; wt, wild‐type; yk, transgenic mice with induced Yamanaka factor expression.

### 3.4 Partial Reprogramming Prevents the Loss of Myelin pMCAo‐Induced

One of the pathological features after an ischemic event in both humans and rodents is demyelination (Li et al. 2024; Martín‐Lopez et al. 2024). White matter alterations have been reported following cerebral ischemia (Barker et al. 2013; Martín‐Lopez et al. 2024). To determine whether partial reprogramming affected the loss of myelin after pMCAo, we selected six adjacent fields (see Figure 5D) from each brain slice stained with Black‐Gold to calculate the percentage of positive area (Figure 5A,E). We found no significant difference between the two genotypes in the Sham groups, suggesting that cyclic induction of YFs for 4 weeks, in the absence of ischemia, does not affect the degree of myelination of the tissue. As expected, we found a drastic reduction of the percentage of Black‐Gold positive area in pMCAo‐wt compared to the Sham group (61.7% ± 10.42%; *p* ≤ 0.05), a clear indicator of demyelination after ischemia (Figure 5A,E). However, the Black‐Gold staining did not disclose any significant difference between Sham‐yk and pMCAo‐yk groups, indicating that OSKM induction after pMCAo preserved myelin after 48 h of ischemia (Figure 5A,E). However, our results show a significant difference between ischemic groups (61.7% ± 10.42%; pMCAo‐wt and 108% ± 9.03% pMCAo‐yk; *p* ≤ 0.05).

**FIGURE 5:**
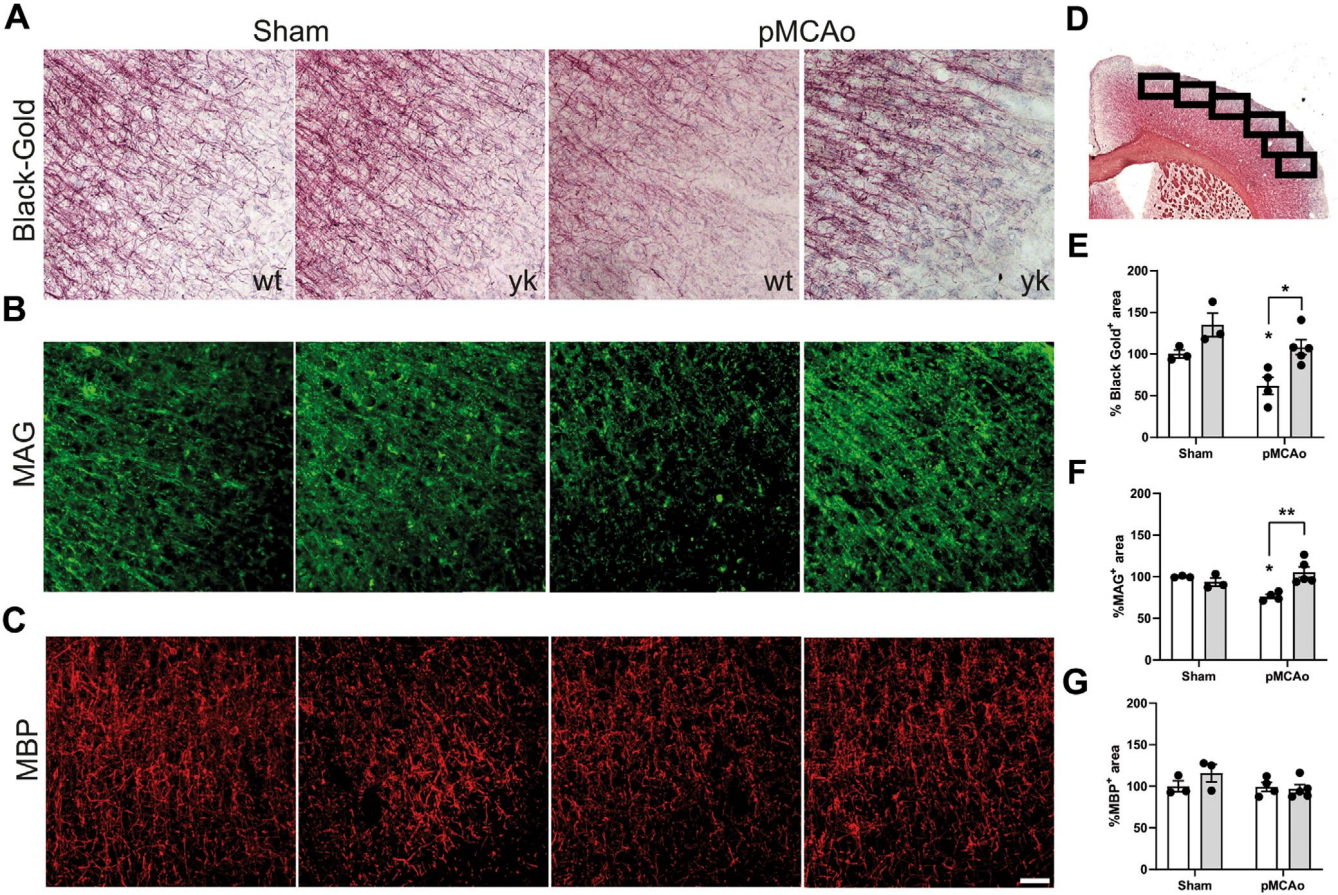
Partial reprogramming attenuates pMCAo‐induced myelin loss. (A) Representative Black Gold II–stained images of the parietal cortex from Sham‐operated and pMCAo mice. (B, C) Representative immunofluorescence images of the parietal cortex from sham and pMCAo mice stained for MAG (green; B) and MBP (red; C). Scale bar: 20 μm. (D) Low‐magnification image of a Black Gold II–stained coronal section of the right parietal cortex; boxes indicate the six fields of interest used for quantification in each brain section. (E–G) Quantification of the percentage of positive area for Black Gold II (E), MAG (F), and MBP (G) staining in Sham and pMCAo groups. Data are presented as mean ± SEM (*n* = 3 in sham groups, *n* = 4 in pMCAo‐wt, and *n* = 5 in pMCAo‐yk). White bars correspond to wt and the gray bars to yk genotypes. Statistical significance was determined by two‐way ANOVA with Bonferroni *post hoc* correction. *p < 0.05; **p < 0.01. MAG, myelin‐associated glycoprotein; MBP, myelin basic protein; pMCAo, permanent middle cerebral artery occlusion; wt, wild‐type; yk, transgenic mice with induced Yamanaka factor expression.

To confirm these results, we analyzed two myelin‐associated proteins: myelin‐associated glycoprotein (MAG) and myelin basic protein (MBP) (Figure 5B,C). MBP levels were similar in all experimental groups (Figure 5C,G). However, the levels of MAG in pMCAO‐wt mice decreased significantly to 76.3% ± 2.47% compared to the Sham‐wt group (Figure 5B,F) (*p* ≤ 0.05). Conversely, there were no significant differences in pMCAo‐yk and Sham‐yk experimental groups. These findings strongly indicate that the cyclic overexpression of YF helps to prevent the loss of myelin induced by ischemia.

### 3.5 Cyclic Induction of YK Factors Promotes a Significant Increment in the Density of Olig2^+^ Cells in the Damage Area 48 h After pMCAo

Oligodendrocytes are the cells responsible for myelination. It has been described that oligodendrocytes respond to ischemia by colonizing the damaged area to maintain axon integrity long‐term (Chida et al. 2011; Liu et al. 2019; Villa González et al. 2020). Since the induction of YFs in transgenic mice helped maintain myelin integrity after pMCAo induction, we analyzed the response of oligodendrocytes 48 h after damage. To assess the variation of oligodendrocyte populations in the damaged area, we stained brain slices with specific markers of precursors (OPCs; PDGFRα^+^ cells, Figure 6A,C), pre‐myelinating (BCAS1^+^; Figure 6B,F), mature (CC1^+^ cells; Figure 6C,G), and total oligodendrocytes (Olig2^+^ cells; Figure 6D,H). We quantified the cell density of each population in brain sections obtained from Sham and pMCAo mice of both genotypes (wt and yk). Our findings indicated that the 4‐week induction of the Yamanaka factor did not affect the Sham animals, as both genotypes exhibited similar values across all analyzed populations. However, total oligodendrocytes/mm^2^ (Olig2^+^ cells) increased following 48 h of pMCAo, being significantly greater in transgenic animals (338 ± 21 cells/mm^2^ in pMCAo‐wt and 497 ± 33 cells/mm^2^ in pMCAo‐yk; *p* < 0.01) (Figure 6D,H). The density of OPCs (PDGFRα^+^ cells/mm^2^) significantly increases after 48 h of pMCAo in yk mice, reaching a value of 80.02 ± 3.12 cells/mm^2^, compared to Sham‐yk (51.17 ± 2.88 cells/mm^2^; *p* < 0.001) (Figure 6A,E). Density of mature oligodendrocytes (CC1^+^ cells/mm^2^; Figure 6C,G) showed no significant differences in any experimental group. The population of pre‐myelinating oligodendrocytes (BCAS1^+^ cells/mm^2^; Figure 6B,F) increased after 48 h of pMCAo in the wt group, reaching a value of 303.7 ± 34.1 cells/mm^2^ (164.41 ± 7.62 cells/mm^2^ on the Sham‐wt group; *p* < 0.05).

**FIGURE 6:**
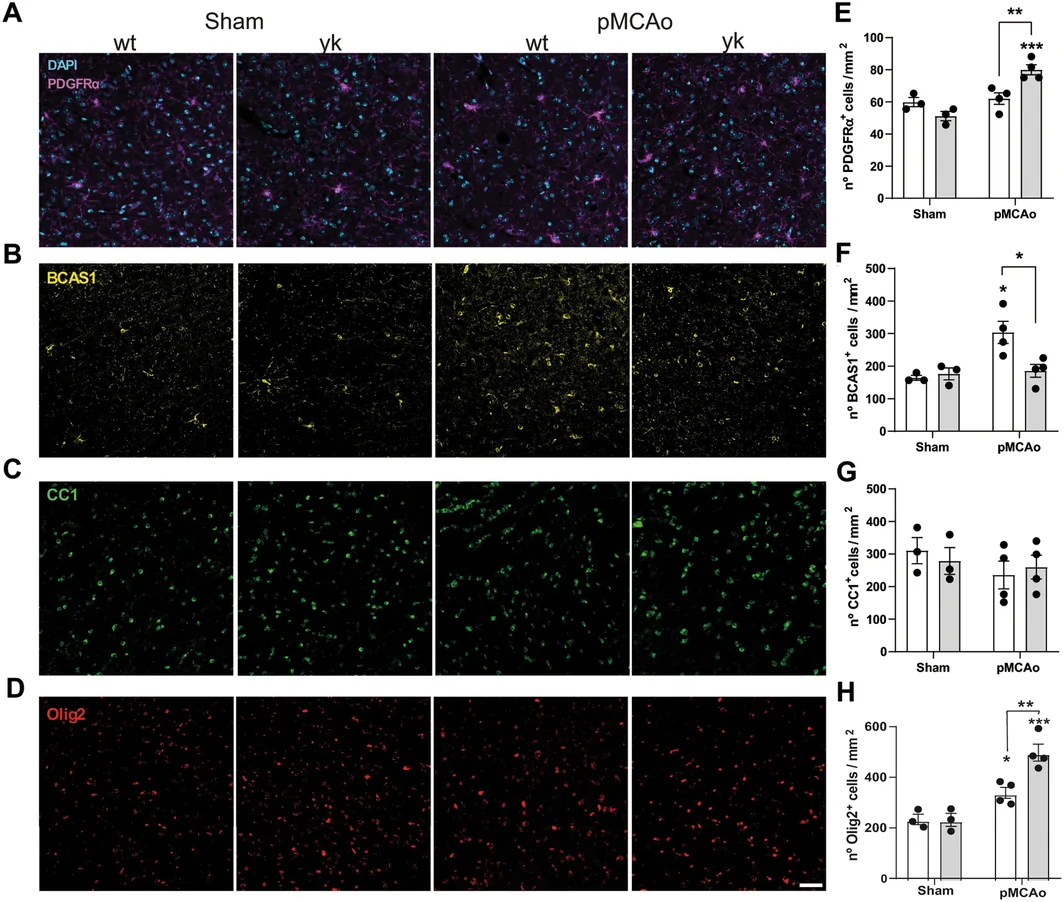
Effect of partial reprogramming on oligodendrocyte subpopulations in the damaged area after cerebral ischemia. (A–D) Representative confocal images of the parietal cortex from coronal brain sections (25 μm thick) of sham‐operated and pMCAo mice stained with DAPI (cyan) and markers of oligodendrocyte lineage stages: PDGFRα (magenta) for oligodendrocyte precursor cells (A), BCAS1 (yellow) for premyelinating oligodendrocytes (B), CC1 (green) for mature oligodendrocytes (C), and Olig2 (red) for the total oligodendrocyte lineage (D). Scale bar: 20 μm. (E–H) Quantification of cell density (cells/mm^2^) for PDGFRα^+^ (E), BCAS1^+^ (F), CC1^+^ (G), and Olig2^+^ (H) cells in sham and pMCAo mice of each genotype. Individual data was obtained using three FOVs per brain section from each mouse. The white bars correspond to wt and the gray bars to yk genotype. Data are presented as mean ± SEM (*n* = 3 in sham groups, *n* = 4 in pMCAo groups). Statistical analysis was performed using two‐way ANOVA with Bonferroni *post hoc* correction. *p < 0.05; **p < 0.01; ***p < 0.001. BCAS1, breast carcinoma–amplified sequence 1; CC1, adenomatous polyposis coli (APC); Olig2, oligodendrocyte transcription factor 2; PDGFRα, platelet‐derived growth factor receptor alpha; pMCAo, permanent middle cerebral artery occlusion; wt, wild‐type; yk, transgenic mice with induced Yamanaka factor expression.

## 4 Discussion

OSKM transcriptional factors (Oct4, Sox10, Klf4, and Myc) were identified by Yamanaka in 2006 (Takahashi and Yamanaka 2006). Their overexpression in vitro can induce reprogramming of differentiated adult cells into pluripotent stem cells (iPSCs). Later, in vivo cyclic overexpression of YK factors, known as partial cell reprogramming, demonstrated an effective approach to cell “rejuvenation” without total dedifferentiation (Abad et al. 2013; Ocampo et al. 2016; Sichani et al. 2024).

Brain aging is associated with the accumulation of both molecular and cellular damage that reduces cognitive functions and increases vulnerability to other neurodegenerative disorders, such as stroke (Cipriano et al. 2024; Roy‐O'Reilly and McCullough 2018; Springer et al. 2024). Multiple molecular hallmarks have been identified in the aging nervous system by transcriptomics and proteomic studies, including mitochondrial dysfunction, loss of stem cell function, cellular senescence, loss of proteostasis, DNA damage, among others (Cipriano et al. 2024; Huang et al. 2023). Epigenetic changes based on DNA methylation emerged as an indicator of tissue aging, owing to their association with several neuropathological hallmarks of Alzheimer's disease and with cognitive decline (Cipriano et al. 2024; Levine et al. 2015). Partial cell reprogramming reduces or eliminates epigenetic marks related to aging, while maintaining cellular identity, reversing or mitigating aging‐associated decline, as has been demonstrated in some mammalian organs of premature aging mice (Browder et al. 2022; Sichani et al. 2024); however, the effects on the brain remain largely unexplored (Antón‐Fernández et al. 2024; Rodríguez‐Matellán et al. 2020; Sarkar et al. 2020; Shen et al. 2024; Sichani et al. 2024).

Until now, the available studies related to in vivo partial reprogramming in the brain have been performed in adult animals (2–7 months old), using a transient induction of YFs for 5 months (Antón‐Fernández et al. 2024; Antón‐Fernández et al. 2025; Rodríguez‐Matellán et al. 2020; Shen et al. 2024). In other tissues, partial reprogramming using in vivo cyclic overexpression of OSKM, in old animals (12–25 months old), has been demonstrated with an induction protocol of 7–10 months, preventing epigenetic changes associated with aging in skin and kidney (Browder et al. 2022). Our protocol of cyclic administration of doxycycline over a period of 4 weeks led to a marked increase in both the distance traveled and speed during the open field test, alongside a reduction in total resting time in yk‐mice compared to wt‐mice, indicative of enhanced motor capacity. Previous reports have shown a reduction in motor activity in aging mice using the open field test (Radulescu et al. 2021). Therefore, this improvement in motor capacity could be an indicator of brain rejuvenation. Notably, the yk‐mice exhibited no significant increase in anxiety, as evidenced by their time spent in the center of the arena being comparable to that of wt‐mice. Similar results have been recently obtained by inducing YFs during brain development (E10.5 to E14.5), which resulted in cortical expansion and an improvement in motor behavior at 5‐month‐old adult mice (Browder et al. 2022). Our results indicate that 4 weeks of doxycycline administration is sufficient to enhance motor capacity in yk‐mice. While this improvement could be compatible with brain rejuvenation, additional studies will be required to determine whether rejuvenation mechanisms underlie the observed effects. Importantly, such benefits remain attainable in aged animals.

The transgenic mouse line used in this study contains a doxycycline‐inducible polycistronic cassette that encodes the four Yamanaka reprogramming factors, which are expressed ubiquitously. As a result, the overexpression of OSKM factors following doxycycline administration (measured by Oct4^+^ and Klf4^+^ cells) was observed in neurons, microglia, astrocytes, and oligodendrocytes, which makes this a useful model to investigate the effects of cellular reprogramming of all cellular types in the aging brain, rather than focusing on reprogramming within a specific cell type. Notably, this is the first time the overexpression of YFs in oligodendrocytes and microglia has been described in vivo beyond neurons and astrocytes (Antón‐Fernández et al. 2025; Shen et al. 2024). These findings strongly suggest that all cellular populations in the brain in our yk‐mice are equally susceptible to partial reprogramming in vivo.

Despite widespread OSKM overexpression across brain cell types, our results indicate that this approach did not alter neurogenesis or gliogenesis in the aging brain, as no differences in the cellular densities of major cell populations were detected in yk‐mice compared with wt‐mice. These findings are consistent with previous studies reporting no significant differences in astrocyte or neuronal abundance in the adult mouse hippocampus (Antón‐Fernández et al. 2025) and in contrast to findings obtained following YF overexpression during brain development (Browder et al. 2022). Our results support the possibility that YF overexpression in aged mice promotes features consistent with a more youthful brain phenotype without detectable effects on cell proliferation, including that of glial cells, which retain proliferative potential in the adult brain. These findings suggest that partial reprogramming may represent a potential strategy to mitigate certain effects of brain aging through improvements in cellular function while avoiding the induction of excessive proliferation.

Neurodegenerative diseases, such as stroke, occur with significantly high incidence among the older population (Roy‐O'Reilly and McCullough 2018; Springer et al. 2024). Stroke is the second cause of mortality and the first cause of long‐term disability worldwide, with a high incidence in the elderly and poor prognosis related to patient age (Martin et al. 2025). After confirming that partial reprogramming in aging mice improves their motor capabilities, we wanted to know if it could protect against the harmful effects of brain ischemia.

One of the pathological indicators of ischemic damage is the reduction of neuronal density. Our results show that while partial reprogramming for 4 weeks does not affect neuronal density of yk‐mice, it does confer protection against ischemic injury, as the notable reduction observed in pMCAO‐wt mice after 48 h of ischemia is not observed in pMCAO‐yk mice, likely due to the effects of YF overexpression. Although the mechanism underlying these effects remains unclear, one possibility is that partial reprogramming induces metabolic adaptations in neurons. In this regard, we speculate that partial reprogramming could facilitate a shift from oxidative phosphorylation to glycolysis/ketogenesis, similar to what has been observed in other excitable cells, such as cardiomyocytes (Cheng et al. 2022). If present, such a metabolic shift could reduce neuronal dependence on oxygen and potentially enhance resilience under ischemic conditions. However, this hypothesis was not directly assessed in the current study and will require further investigation.

This neuronal loss observed in pMCAO‐wt after ischemia triggers a glial cell response, observed as an increase in GFAP^+^ cell density in the penumbra region, absent in the pMCAO‐yk group. On the other hand, 48 h after pMCAo, there is no significant increase in microglial cell density in either ischemic group compared to the Sham, as microglial response to ischemic damage is an early and dynamic process, peaking within 24 h of ischemia onset (Hernández et al. 2021; Rupalla et al. 1998; Villa‐González et al. 2024). Collectively, these results suggest that cyclic overexpression of YFs provides a protective effect against ischemic injury by preserving neuronal viability and ameliorating reactive glial response.

Another pathological indicator of ischemic damage is the loss of myelin, which results from injury by both neurons and oligodendrocytes (Aboul‐Enein et al. 2003; Cheng et al. 2024; Hernández et al. 2021; Martín‐Lopez et al. 2024; Reimer et al. 2011). In addition, demyelination exacerbates neuronal injury by compromising axonal protection and increasing their vulnerability to degeneration (Martín‐Lopez et al. 2024; Shi et al. 2015). Consequently, strategies focused on myelin preservation or remyelination have proven effective for reducing ischemic damage (Martín‐Lopez et al. 2024; Shi et al. 2015). Our results demonstrated that partial reprogramming in Sham yk‐mice did not affect the total myelin, MBP, and MAG compared to wt. However, pMCAo induced a significant reduction in total myelin in wt‐mice, in accordance with previous reports in humans and rodents (Aboul‐Enein et al. 2003; Cheng et al. 2024; Martín‐Lopez et al. 2024). The last is accompanied by a significant diminution in MAG‐stained and no variation in the MBP‐stained area compared to the Sham groups. MAG, a glycoprotein located distally on periaxonal processes, anchors the oligodendrocyte membrane to the axon and is affected by ischemic damage earlier than MBP (Aboul‐Enein et al. 2003; Barker et al. 2013). The observed loss of MAG and non‐MBP in wt mice following 48 h of pMCAo induction may suggest an early trend of myelin degeneration, as supported by our Black gold staining results, which could indicate an early disruption of axon‐glia integrity at this timepoint (Reimer et al. 2011). However, our results suggest that partial reprogramming provides a protective effect against ischemic damage, maintaining myelin integrity as pMCAo‐yk mice maintained similar levels to the Sham of both Black gold and MAG after pMCAo. The maintenance of the %MAG^+^ area in pMCAo‐yk mice, like the sham group, suggests improved axon‐oligodendrocyte pairing after OSKM induction, which might confer resilience to ischemia‐induced demyelination. These results align with other reports that relate aging to a greater susceptibility to: (i) demyelination after ischemic damage (Miyamoto et al. 2013); (ii) a reduced regenerative capacity of myelin fibers in the cerebral cortex; and (iii) decline of remyelination efficiency after damage (de la Fuente et al. 2020; Radulescu et al. 2021).

YK overexpression could lead to the generation of neurons that exhibit resistance to hypoxic conditions (Simon and Keith 2008) or enhance the resilience of oligodendrocytes against ischemic damage, thereby helping to preserve myelin and consequently axon integrity, which improves neuron viability. To explore this further, we analyzed the effects of OSKM induction on the density of total oligodendrocytes (Olig2^+^ cells/mm^2^) as well as on the specific population of oligodendrocytes: OPCs (PDGFRα^+^ cells), premyelinating (BCAS1^+^ cells), and mature (CC1^+^ cells). Our findings indicate that OSKM induction does not lead to significant differences in the density of total oligodendrocytes or within any specific population in Sham‐operated animals. However, after ischemia, we observed an increase in oligodendrocyte density in the affected area in wt‐mice, consistent with previous studies (Chida et al. 2011; Martín‐Lopez et al. 2024). Concurrently, the population of pre‐myelinated oligodendrocytes in pMCAo‐wt also increased, which could indicate an early remyelination process triggered by ischemic demyelination (Martín‐Lopez et al. 2024). Interestingly, the oligodendrocyte response in the pMCAo‐yk group was notably greater than in wild‐type mice, as indicated by a significant rise in Olig2^+^ cells and OPCs density in the damaged area. These findings suggest that OSKM induction enhanced the recruitment of OPCs and oligodendrocytes to the injured area, resembling responses previously described in younger brains (Miyamoto et al. 2013). These recruited OPCs could differentiate into mature oligodendrocytes to restore myelin later and may also participate in the inflammatory response, as evidenced by their capacity to differentiate into astrocytes (Chen et al. 2020).

CC1^+^ oligodendrocytes exhibited similar levels to the Sham group across both pMCAo experimental groups (wt and yk). This observation likely results from the timing of the analysis, which was conducted during the acute phase of ischemia, since it has been documented that oligodendrocyte maturation occurs at a later stage (Martín‐Lopez et al. 2024).

All in all, partial reprogramming demonstrates a protective effect against ischemic injury, maintaining neuronal viability, myelin integrity, and mitigating glial responses in the affected area 48 h following the onset of pMCAo, which is typically compromised in the wild‐type mice. This was reflected in the reduction in tissue damage and in the partial improvement of motor functions of pMCAO‐yk animals in comparison to pMCAO‐wt, similarly to what has been described previously using neuroprotectant agents (Jia et al. 2024; Mateos et al. 2016; Pérez‐Álvarez et al. 2012) and strategies focused on myelin preservation or remyelination that are effective for reducing ischemic damage (Martín‐Lopez et al. 2024; Shi et al. 2015). The lack of significant differences in the beam walking tests may reflect the limited sensitivity of those tasks to detect deficits associated with acute parietal cortical injury (Chen et al. 2019). Notably, partial cellular reprogramming does not alter the density of any cell type under normal conditions; therefore, the observed enhancement in motor function is likely attributed to improved functionality of the existing cells and neuronal circuits.

In our mouse model, it is plausible that there may be partial reprogramming of the endothelial cells within the brain's microvessels. It has been noted that aging affects the integrity of the blood–brain barrier (BBB), leading to increased permeability due to enhancement in paracellular flux (Kim and Cheon 2024; Stamatovic et al. 2019; Ya et al. 2023). Dysfunction of the BBB exacerbates ischemic damage, being one of the pathophysiological features of ischemic stroke (Abdullahi et al. 2018; Sasannia et al. 2025). We cannot dismiss the possibility that partial reprogramming could enhance the resilience of brain endothelial cells to ischemia‐induced permeability, ultimately protecting neural cells from damage. Recently, it has been described that partial reprogramming of endothelial cells can alleviate senescence, reduce inflammatory response, and improve extracellular matrix organization, thereby ameliorating the detrimental effects of hypertension (Pernomian et al. 2025), a condition associated with aging (Sun 2015).

Finally, our data shows that controlled cyclic overexpression of YFs in aging mice over 4 weeks is associated with neuroprotective effects following brain ischemia, a condition for which aging is a major risk factor and is linked to poorer outcomes. These findings suggest that this approach may help attenuate the impact of stroke in the brain, as reflected by improved neurological scores and reduced myelin and neuronal damage in mice. Although our model does not allow the observed benefits to be attributed to a single cellular population, since both glial cells and neurons show evidence of YF overexpression, the results raise the possibility that partial reprogramming may contribute to beneficial responses following stroke.

This study has several limitations that should be considered. First, although both male and female mice were included, the sample size was not designed to allow a statistically powered sex‐specific analysis, and therefore, potential sex‐dependent effects of YF overexpression cannot be excluded. Second, the analyses were restricted to the acute phase following ischemia (48 h post‐pMCAO), preventing assessment of the long‐term functional and histological consequences of the intervention. Third, the potential effect of doxycycline on the observed phenotype. As a broad‐spectrum antibiotic, doxycycline administration can lead to several biological effects, including gut microbiota dysbiosis (Boynton et al. 2017), neuroprotective effect against inflammatory insults (Yrjänheikki et al. 1998), impair mitochondrial function (Wüst et al. 2020), and also can alter baseline gene expression (Sultan et al. 2013). These effects may differ between wt and transgenic strains. However, doxycycline‐free controls are redundant because non‐induced Tet‐On transgenic mice are functionally identical to wt mice. Thus, uniform doxycycline exposure ensures that observed phenotypic changes are driven purely by genetic activation.

## Author Contributions

P.R.M. and G.M.‐L. have contributed equally. Formal Analysis, investigation, methodology, visualization, writing review, and editing. J.R.‐M. methodology, investigation, visualization, and writing review. J.Á. Conceptualization and writing review. F.H. Conceptualization, writing review, funding acquisition, validation, project administration. M.J.P.‐Á. Conceptualization, data curation, funding acquisition, validation, project administration, supervision, writing‐original draft, writing review and editing.

## Funding

This work was supported by Ministerio de Ciencia, Innovación y Universidades, PID2023‐149460NB‐I00, PID2020‐115876GB‐I00, Fundación Ramón Areces, CIVP22A7607, Fundación Tatiana Pérez de Guzmán el Bueno, and Departamento de Biologia‐Facultad de Ciencias UAM, BIOUAM02‐2024.

## Conflicts of Interest

The authors declare no conflicts of interest.

## Supporting information


**Figure S1:** Cyclic doxycycline administration induces Yamanaka factor expression across major neural cell types. (A, B) Representative confocal images of the parietal cortex from coronal brain sections (25 μm thick) of wt and yk mice, following cyclic doxycycline treatment, stained for Klf4 (A), and Oct4 (B), confirming induction of Yamanaka factors. Scale bar: 20 μm. (C, D) Representative confocal images of the parietal cortex from coronal brain sections (25 μm thick) of yk mice, following cyclic doxycycline treatment, immunostained for Klf4 (C) or Oct4 (D) and cell type–specific markers (green): Olig2 for oligodendrocytes, NeuN for neurons, GFAP for astrocytes, and Iba1 for microglia. Orthogonal views confirm marker colocalization. Scale bar: 5 μm. Klf4, Krüppel‐like factor 4; Oct4, octamer‐binding transcription factor 4; Olig2, oligodendrocyte transcription factor 2; NeuN, neuronal nuclei; GFAP, glial fibrillary acidic protein; Iba1, ionized calcium‐binding adapter molecule 1.

**Table S1:** Sex, genotype, and age (start doxycycline treatment) of the mice used for each experiment.

## Data Availability

The raw will be available in the public repository from Universidad Autónoma de Madrid/Consorcio Madroño: eciencia datos (https://edatos.consorciomadrono.es/): https://doi.org/10.21950/MZT0EH.
