# Supplementary material for: In Vivo Partial Cell Reprogramming With Yamanaka Factors Reduced Ischemic Brain Damage in Aging Mice
Source: Aging Cell. 2026 Oct 23;25(11):e70776. doi: 10.1111/acel.70776 (PMC13692703; doi:10.1111/acel.70776)
Supplement: Supplementary file 1 [file ACEL-25-e70776-s002.docx]

**Table 1**: Sex, genotype, and age (start doxycycline treatment) of the mice used for each experiment.
